# Differential outcome of concurrent radiotherapy plus epidermal growth factor receptor inhibitors versus radiotherapy plus cisplatin in patients with human papillomavirus-related head and neck cancer

**DOI:** 10.1186/1471-2407-13-26

**Published:** 2013-01-18

**Authors:** Bella Pajares, Jose M Trigo, Maria D Toledo, Martina Álvarez, Carlos González-Hermoso, Antonio Rueda, Jose A Medina, Vanessa de Luque, Jose M Jerez, Emilio Alba

**Affiliations:** 1 Department of Medical Oncology, University Hospital Virgen de la Victoria, Málaga, Spain; 2 Department of Radiotherapy, University Hospital Virgen de la Victoria, Málaga, Spain; 3 Department of Pathology, University Hospital Virgen de la Victoria, Málaga, Spain; 4 Hospital Costa del Sol, Department of Pathology, Marbella, Málaga, Spain; 5 Hospital Costa del Sol, Department of Medical Oncology, Marbella, Málaga, Spain; 6 Biomedical Research Laboratory Hospital Virgen de la Victoria, Málaga, Spain; 7 Computational Languages Department, Málaga University, Málaga, Spain

**Keywords:** Head and neck cancer, Human papillomavirus, Chemotherapy, Radiotherapy, EGFR inhibitors

## Abstract

**Background:**

Human papillomavirus (HPV)-related head and neck cancer has been associated with an improved prognosis in patients treated with radiotherapy (RT) +/− chemotherapy (CT); however, RT combined with epidermal growth factor receptor (EGFR) inhibitors has not been fully studied in this group of patients.

**Methods:**

Immunohistochemical expression of p16 and PCR of HPV16 DNA were retrospectively analyzed in tumor blocks from 108 stage III/IV head and neck cancer patients treated with RT+CT (56) or RT+EGFR inhibitors (52). Disease-free survival (DFS) and overall survival (OS) were analyzed by the Kaplan-Meier method.

**Results:**

DNA of HPV16 was found in 12 of 108 tumors (11%) and p16 positivity in 18 tumors (17%), with similar rates in both arms of treatment. After a median follow-up time of 35 months (range 6–135), p16-positive patients treated with RT+EGFR inhibitors showed improved survival compared with those treated with RT+CT (2-year OS 88% vs. 60%, HR 0.18; 95% CI 0.04 to 0.88; p = 0.01; and 2-year DFS 75% vs. 47%, HR 0.17; 95% CI 0.03 to 0.8; p = 0.01). However, no differences were observed in p16-negative patients (2-year OS 56% vs. 53%, HR 0.97; 95% CI 0.55 to 1.7; p = 0.9; and 2-year DFS 43% vs. 45%, HR 0.99; 95% CI 0.57 to 1.7; p = 0.9).

**Conclusions:**

This is the first study to show that p16-positive patients may benefit more from RT+EGFR inhibitors than conventional RT+CT. These results are hypothesis-generating and should be confirmed in prospective trials.

## Background

Head and neck squamous cell carcinoma (HNSCC) is the sixth most common cancer worldwide, with an estimated annual burden of 633,000 incident cases and 355,000 deaths [1]. This neoplasm is largely attributed to environmental exposures, such as tobacco and alcohol consumption [2]. However, a subset of HNSCC, specifically oropharyngeal squamous cell carcinomas (OPSCCs) located in the base of the tongue and in the tonsils, and less frequently oral cavity and hypopharynx squamous cell carcinomas, may occur in non-smokers and non-drinkers, suggesting the presence of other risk factors. Recent epidemiological and molecular studies suggest that human papillomavirus (HPV) infection, the necessary cause of cervical carcinoma, is involved in the pathogenesis of a subset of these neoplasms [3-7].

HPV genomic DNA has been found in approximately 20-25% of all HNSCCs using sensitive polymerase chain reaction (PCR)-based methods, with a greater prevalence in OPSCC (36-75%) [4,8-11], and p16^INK4A^ (p16) overexpression has also been correlated with HPV positivity [12-16].

Several studies, including retrospective cases series, retrospective analyses of prospective studies and phase III trials, have shown that patients with HPV-related HNSCC managed with radiotherapy (RT) +/− chemotherapy (CT) have better prognosis compared with patients with HPV-negative tumors in terms of response and survival [13,14,17-21]. This benefit has also been observed in p16-positive patients compared with p16-negative patients [14,21-24]. Moreover, a recent meta-analysis with more than 5,600 patients from 34 studies showed a better prognosis in terms of survival for HPV-positive HNSCC (HR, 0.42; 95% CI 0.27 to 0.57; p < 0.0001), specially in OPSCCs (HR, 0.4; 95% CI 0.18 to 0.61; p < 0.0001) [25]. All these studies involved patients treated with different protocols, including different combinations of RT and CT.

Over the past decade, clinical research on HNSCC has focused on improving the efficacy of current multimodal approaches and decreasing toxicity by targeting cellular pathways associated with carcinogenesis. Blocking the epidermal growth factor receptor (EGFR) has emerged as a primary strategy, although not much information is available about these therapies in HPV-positive patients. In the present study, we aimed to retrospectively evaluate the impact of p16 expression and HPV16 DNA positivity on response and survival in patients with HNSCC treated with a combination of RT plus EGFR inhibitors compared with patients treated with RT+CT.

## Materials and methods

### Patient data and specimen characteristics

Between 2000 and 2011, 116 patients with newly diagnosed locally advanced HNSCC (stage III and IV non-metastatic) who were candidates for radical RT combined with CT or EGFR inhibitors were treated under different protocols in our center. A total of 108 patients were fully assessable in terms of availability of pathological specimens. Baseline studies included physical examination, chest X-rays, endoscopy of the upper aerodigestive tract and computed tomography of the neck. The response to the treatment was assessed 6–8 weeks after the end of therapy by RECIST criteria. After treatment, all patients underwent clinical examinations and imaging on a regular basis. We also assessed reliable information about tobacco exposure and alcohol consumption. Patients were assessed for the occurrence of HNSCC relapse, second tumors (ST) and death. ST was clinically defined as a tumor occurring more than 2 centimeters away and more than 3 years after the treatment of the primary tumor. Fifty-six patients received concurrent RT plus platinum-based CT, and 52 patients received several types of EGFR inhibitors, mainly cetuximab, concurrent with RT.

CT was administered as three-weekly cisplatin doses of 100 mg/m^2^ (32 pts.) or weekly cisplatin at 40 mg/m^2^ (24 pts.). EGFR inhibitors were mainly administered as weekly cetuximab at an initial dose of 400 mg/m^2^ followed by 250 mg/m^2^ (36 pts.), three-weekly panitumumab at 9 mg/kg (11 pts.) or daily gefitinib at 250 mg/day (5 pts.). Radiation treatment was delivered with curative intention to the whole series by the technique of three-dimensional conformal radiotherapy. The median dose of radiotherapy was 72 Gy (57–78 Gy). Ninety-seven patients received accelerated fractionation with concomitant boost, and 11 patients were treated with standard fractionation. Standard fractionation was administered in 1.8 or 2 Gy per fraction, 5 fractions a week to 70 Gy in 35 fractions over 7 weeks. Accelerated fractionation with concomitant boost was delivered in 1.8 Gy per fraction, 5 fractions a week to 54 Gy in 30 fractions over 6 weeks, to the initial target volume encompassing the gross tumor and clinically/radiologically involved nodes along with regions of potential contiguous and lymphatic spread. At 32.4 Gy, a second daily dose of 1.5 Gy per fraction (with an interval of at least 6 hours) was given to the boost volume covering the gross tumor and involved nodes for a total of 18 Gy in 12 treatment days. The primary tumor and clinically/radiologically involved nodes received 72 Gy in 42 fractions over 6 weeks, and uninvolved nodes received 54 Gy over 6 weeks. During the treatment, patients were seen at least weekly by a radiation oncologist or medical oncologist and more frequently when necessary. The median follow-up time was 35 months (range 6–135).

### Statistical analysis

Patients, toxic habits, type of treatment and disease characteristics were tabulated by means of frequency tables. Qualitative variables are expressed as a percentage with a 95 confidence interval of the percentage, and quantitative variables are expressed as the median and range. Variable comparisons were carried out using the Wilcoxon or chi-square tests. The end points of interest were overall survival (OS), disease-free survival (DFS) and tumor relapse. OS was defined as the time from first treatment to death due to any cause. DFS was defined as the time from first treatment to first documented relapse, secondary tumor or death by any cause. Tumor relapse was defined as head and neck cancer recurrence any time after RT+CT treatment. To investigate the pattern of occurrence over time of any of the aforementioned end points, descriptive analyses were carried out by estimating Kaplan-Meier survival curves, whereas inferential analyses relied on cumulative hazards. In particular, unadjusted p values for testing the prognostic effect of HPV16 DNA and p16 overexpression status were obtained from the log rank test, and adjusted p values were obtained from the likelihood ratio test in a multivariable Cox regression model. p values below the conventional 5% threshold were regarded as significant. All the analyses were carried out using R and SPSS version 15.0 software.

### p 16 Immunohistochemistry

p16 immunohistochemistry (IHC) was performed on formalin-fixed, paraffin-embedded (FFPE) tissue sections using a CINtec® Histology Kit (mtm laboratories AG, Germany) for the qualitative detection of the p16 (antibody clone E6H4 TM) in a Dako autostainer (Dako, Copenhagen, Denmark). Cervical cancer sections known to be p16 positive were used as positive controls, and omission of primary antibody was used as the negative control. All p16 IHC biopsies were semiquantitatively scored by a pathologist blinded to the patient’s HPV status. Tumors were classified dichotomously as either p16-positive (strong, diffuse staining) or p16-negative.

### HPV Polymerase chain reaction

FFPE tissue sections were assessed by a pathologist to determine the percentage of tumor in each section. DNA was extracted using a DNA Blood mini kit (Promega). HPV was detected by PCR by using a PapType human papillomavirus detection test (Kit Screening HPV, Master Diagnostica S.L.) using the primer Gp 5-6/L1. Then, the positive cases were genotyped to detect the specific HPV subtype (HPV GenoArray Test Kit, Master Diagnóstica S.L.). This assay detects 17 subtypes of high-risk HPV (16, 18, 31, 33, 35, 39, 45, 51, 52, 53, 56, 58, 59, 66, 68, 73 and 82) and 16 low-risk HPV subtypes (6, 11, 40/61, 42, 43/44, 54/55, 70, 57/71, 72, 81, and 84/26). All samples were amplified in duplicate to provide a control of method reproducibility.

### Ethics statement

This study was carried out in compliance with the Declaration of Helsinki (http://www.wma.net/en/30publications/10policies/b3/index.html). All subjects provided informed consent for study inclusion, and the study was approved by our hospital's ethics committee (Comité de Etica del Hospital Virgen de la Victoria, Málaga, Spain).

## Results

### Patient characteristics

The vast majority of patients in both groups were male smokers with ECOG 1 performance status and stage IV disease at the time of diagnosis. Twenty-two patients were HPV positive: 17 for high-risk HPV (HPV16, 18, 51 and 58), 3 for low-risk HPV (HPV5 and 6) and 2 for unknown subtypes (Table 1). Twelve of 108 patients were HPV16 positive (11%). Eighteen of 108 cases were p16 positive (17%), and a strong association was found between HPV16 DNA detection and p16 expression. Eight of 12 HPV16-positive tumors exhibited strong and diffuse p16 staining, whereas only 10 of 96 HPV16-negative tumors were p16 positive (67 vs. 10.4%; p < 0.0001) (Table 1). Table 1 shows patient and tumor characteristics according to p16 status. Patients who were p16 positive were less likely to be smokers than p16-negative patients (p < 0.04). The expression of p16 was higher in OPSCCs than in non-OPSCCs (p = 0.04). Table 2 shows patient characteristics according to treatment received. The rate of p16 positivity was similar in both treatment arms: in the group of patients treated with RT+EGFR inhibitors, the rate of positivity was 15% (8/52), and in the group treated with conventional RT+CT, the rate of positivity was 18% (10/56). There were significantly more women and less alcohol consumption in the group of patients treated with RT+EGFR inhibitors compared with the group treated with RT+CT (p < 0.05). On the other hand, there were more patients with an oral cavity location in the group treated with RT+CT (p < 0.05). The proportion of patients treated with standard fractionation was higher in the group treated with EGFR inhibitors compared with those treated with CT (p < 0.003).

**Table 1:** Patient and tumour characteristic by p16 expression status

	**p16 Negative**		**p16 Positive**		
**Patient/Tumour Data**	**No.**	**%**	**No.**	**%**	***p***
**Nº of patients**	**90**		**18**		
**Age, years**					
**Median**	**59**		**57**		**NS**
**Range**	**33-84**		**44-77**		
**Sex**					
**Male**	**84**	**93**	**17**	**94**	**NS**
**Female**	**6**	**7**	**1**	**6**	
**Current smokers**					
**No**	**3**	**3**	**3**	**16**	
**Yes**	**67**	**74**	**10**	**55**	**0.01**
**Unknown**	**20**	**23**	**5**	**29**	
**Alcohol consumption**					
**No**	**10**	**11**	**3**	**16**	
**Yes**	**57**	**63**	**10**	**55**	**NS**
**Unknown**	**23**	**26**	**5**	**29**	
**Performance status**					
**ECOG 0**	**36**	**40**	**9**	**50**	**NS**
**ECOG 1**	**43**	**48**	**8**	**44**	
**ECOG 2**	**11**	**12**	**1**	**6**	
**Tumor site**					
**Oral cavity**	**14**	**16**	**2**	**11**	**0.04**
**Oropharynx**	**30**	**33**	**9**	**50**	
**Larynx**	**36**	**40**	**2**	**11**	
**Hypopharynx**	**10**	**11**	**5**	**28**	
**PCR - HPV status**					
**HR-HPV (16,18,51,58)**	**8**	**9**	**9**	**50**	
**LR-HPV (5,6)**	**2**	**2**	**1**	**5.5**	**0.0001**
**Unknown subtype**	**1**	**1**	**1**	**5.5**	
**Negative**	**79**	**88**	**7**	**39**	
**Grade**					
**Well/Moderate**	**40**	**44**	**6**	**33**	**NS**
**Poor**	**9**	**10**	**2**	**11**	
**Unknown**	**41**	**46**	**10**	**56**	
**Tumour stage**					
**T1-T3**	**37**	**41**	**5**	**28**	**NS**
**T4**	**53**	**59**	**13**	**72**	
**Nodal status**					
**No**	28	31	5	28	**NS**
**N+**	**62**	**69**	**13**	**72**	
**Stage**					
**III**	28	31	6	**33**	**NS**
**IV**	**62**	**59**	**12**	**67**	
**Treatment**					
**RT-CT**	46	**51**	**10**	**56**	**NS**
**RT-EGFR inhibitors**	**44**	**49**	**8**	**44**	
**Irradiation dose**					
**Mean**	**71**		**71.4**		**NS**
**Range**	**57-78**		**70-74**		
**Type of radiotherapy**					
**Conventional RT**	11	12	0	0	**NS**
**Concomitant boost**	**79**	**88**	**18**	**100**	
**Response**					
**Complete response**	67	74	16	89	
**Partial response**	**16**	**18**	**0**	**0**	**NS**
**Stable disease**	**1**	**1**	**1**	**5.5**	
**Progression**	**0**	**0**	**1**	**5.5**	
**Unknown**	**6**	**7**	**0**	**0**	
**Median OS (months)**	**32**		**54**		**NS**
**Range**	**14-50**		**20-87**		
**Median DFS (months)**	**18**		**52**		**NS**
**Range**	**13-24**		**0-103**		

Abbreviation: NS, not significant.

**Table 2:** Patient and tumour characteristic by group of treatment

	**CT-RT**		**RT-EGFRinhibitors**		
**Patient/Tumour Data**	**No.**	**%**	**No.**	**%**	***p***
**Nº of patients**	**56**		**52**		
**Age, years**					
**Median**	58		60		**NS**
**Range**	**44-74**		**33-84**		
**Sex**					
**Male**	55	98	46	89	**0.05**
**Female**	**1**	**2**	**6**	**11**	
**Current smokers**					
**No**	3	5	3	6	**NS**
**Yes**	**28**	**50**	**49**	**94**	
**Unknown**	**25**	**45**	**0**	**0**	
**Alcohol consumption**					
**No**	1	2	12	23	
**Yes**	**28**	**50**	**39**	**75**	**0.02**
**Unknown**	**27**	**48**	**1**	**2**	
**Performance status**					
**ECOG 0–1**	51	91	45	87	**NS**
**ECOG 2-3**	**5**	**9**	**7**	**13**	
**Tumor site**					
**Oral cavity**	13	23	3	6	
**Oropharynx**	**16**	**29**	**23**	**44**	**0.02**
**Larynx**	**17**	**30**	**21**	**40**	
**Hypopharynx**	**10**	**18**	**5**	**10**	
**Grade**					
**Well/Moderate**	34	61	12	23	**NS**
**Poor**	**5**	**9**	**6**	**12**	
**Unknown**	**17**	**30**	**34**	**65**	
**Tumour stage**					
**T1-T3**	20	36	30	58	**NS**
**T4**	**36**	**64**	**22**	**42**	
**Nodal status**					
**N0-N1**	24	43	32	61	**NS**
**N2-N3**	**32**	**57**	**20**	**39**	
**P16 expression**					
**negative**	46	**82**	**44**	**85**	**NS**
**positive**	**10**	**18**	**8**	**15**	
**HPV16 expression**					
**negative**	50	**89**	**46**	**88**	**NS**
**positive**	**6**	**11**	**6**	**12**	
**Irradiation dose**	**71.2**		**71**		**NS**
**Mean**	**57-72**		**70-78**		
**Range**					
**Type of radiotherapy**					
**Conventional RT**	1	2	**10**	**19**	**0.003**
**Concomitant boost**	**55**	**92**	**42**	**81**	
**Response**					
**Complete response**	42	75	41	79	
**Partial response**	**8**	**14**	**8**	**15**	**NS**
**Stable disease**	**2**	**4**	**0**	**0**	
**Progression**	**1**	**2**	**0**	**0**	
**Unknown**	**3**	**5**	**3**	**6**	

### Efficacy

#### Response

The complete response (CR) rates among p16-positive vs. p16-negative cases were 89% and 74%, respectively (OR = 2.7; 95% CI 1.6 to 12.8; p = 0.2) (Table 1). In the 18 patients with p16 expression, those treated with RT+EGFR inhibitors showed a similar CR rate compared with those treated with RT+CT, with CR rates of 100% (8/8) vs. 80% (8/10) respectively; (OR = 2; 95% CI 1.6 to 3.2; p = 0.4). The p16-negative patients showed CR rates of 75% (33/44) when treated with RT+EGFR inhibitors and 74% (34/46) when treated with RT+CT (OR = 1.05; 95% CI 0.4-2.7; p = 1).

#### Recurrences

With a median follow-up time of 35 months (range 6–135), we observed 48 recurrences: 39 locoregional failures and 9 distant failures. Seven patients developed a second tumor (three lung cancer, two gastric cancer, one tongue tumor and one renal tumor). The group of patients with p16 expression showed a 2-year recurrence rate of 28% (5/18), compared to 48% (43/90) among p16-negative patients (HR = 0.4; 95% CI 0,1 to 1.2; p = 0.2). Similar results were also observed for locoregional recurrence, with 2-year locoregional recurrence rates of 22% (4/18) and 39% (35/90),respectively (HR = 0.4; 95% CI 0.1 to 1.4; p = 0.3). These differences were statistically significant for OPSCCs, with a risk of recurrence at 2 years of 11% (1/9) for p16-positive patients vs. 50% (15/30) for p16-negative patients (p = 0.05). In the group of patients with p16-positive tumors, those treated with RT+EGFR inhibitors showed a 2-year recurrence rate of 13% (1/8), which was not significantly different from the 40% (4/10) for those treated with RT+CT (HR = 0.2; 95% CI 0.01-2.4; p = 0.3). p16-negative patients treated with RT+EGFR inhibitors showed a 2-year recurrence rate of 46% (20/44), which was similar to the 50% (23/46) for those treated RT+CT (HR = 0.8; 95% CI 0.4-1.9; p = 0.7).

#### Survival

**Figure 1:**
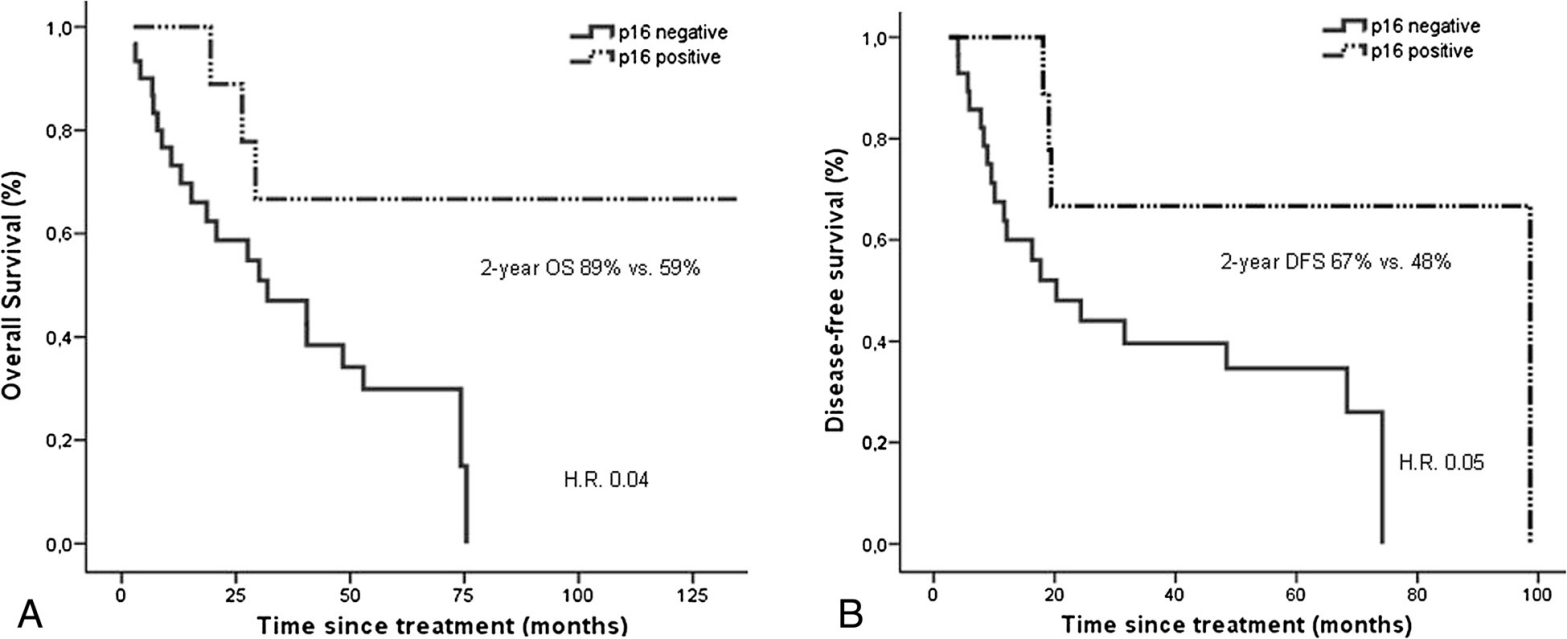
**Kaplan-Meier estimates of survival among OPSCC patients according to p16 expression.** Panels **A** and **B** show the benefits in OS and DFS, respectively, in patients with p16-positive OPSCC compared with p16-negative OPSCC (p = 0.04 and 0.05, respectively).

At the end of follow-up, 67 patients had died: 54 because of primary cancer, 13 because of other causes and 5 patients because of loss to follow-up. Trends for better OS and DFS were observed in the p16-positive group compared with p16-negative patients, with a median OS of 54 vs. 32 months (HR = 0.65; 95% CI 0.3 to 1.2; p = 0.18) and a median DFS of 52 vs.18 months (HR = 0.57; 95% CI 0.28 to 1.1; p = 0.1). These differences were statistically significant in the oropharyngeal location, with 2-year OS rates of 89% for p16-positive patients and 59% for p16-negative patients (HR = 0.3; 95% CI 0.09 to 0.9; p = 0.04) and respective 2-year DFS rates of 67% and 48% (HR = 0.3; 95% CI 0.09 to 0.9; p = 0.05) (Figure 1).

**Figure 2:**
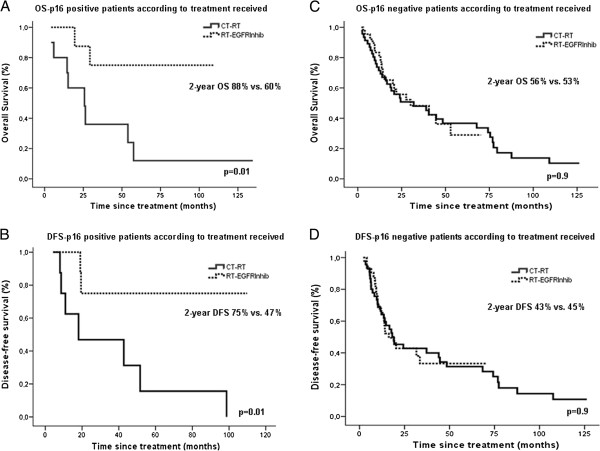
**Kaplan-Meier estimates of survival according to p16 expression and treatment received.** Panels **A** and **B** show OS and DFS, respectively, for p16-positive patients according to the treatment received. Patients with p16-positive tumors treated with RT+EGFR inhibitors had significantly better OS and DFS than those treated with RT+CT (p = 0.01 for both comparisons by the two-sided log-rank test). Panels **C** and **D** show OS and DFS, respectively, for p16-negative patients according to the treatment received. No benefit in OS or DFS was observed in these patients when treated with RT+EGFR inhibitors compared to RT+CT (p = 0.9).

The p16-positive patients treated with RT+EGFR inhibitors showed improved survival compared with those treated with RT+CT (2-year OS: 88% vs. 60%; HR = 0.18, 95% CI 0.04 to 0.88; p = 0.01; 2-year DFS: 75% vs. 47% HR = 0.17, 95% CI 0.03 to 0.8; p = 0.01). However, no survival differences were observed in p16-negative patients treated with RT+EGFR inhibitors compared with those treated with RT+CT (2 year OS: 56% vs. 53%; HR = 0.97; 95% CI 0.55 to 1.7; p =.0.9; 2-year DFS: 43% vs. 45%; HR = 0.99; 95% CI 0.57 to 1.7; p = 0.9) (Figure 2).

Similarly, HPV16-positive patients treated with RT+EGFR inhibitors showed improved survival compared with those treated with RT+CT (2-year OS: 83% vs. 33%; HR = 0.22, 95% CI 0.05 to 0.9; p = 0.02; 2-year DFS: 50% vs. 17%; HR = 0.19, 95% CI 0.47 to 0.8; p = 0.01). However, no survival differences were observed in HPV16-negative patients treated with RT+EGFR inhibitors compared with those treated with RT+CT (2-year OS: 58% vs. 57%; HR = 0.83; 95% CI 0.48 to 1.45; p =.0.5; 2-year DFS: 48% vs. 49%; HR = 0.9; 95% CI 0.53 to 1.5; p = 0.7). In the Cox regression analysis with OS and DFS as the end points, when adjusting for ECOG performance status, tumor size, N category and p16 expression, N category was the only prognostic factor independently associated with a good prognosis in the multivariable analysis (data not shown).

## Discussion

In the present study, we analyzed the association of tumor p16 expression with prognosis in a retrospectively collected cohort of patients with HNSCC treated with RT+EGFR inhibitors or RT+CT. A strong correlation between HPV16 status and p16 immunostaining was found, as well as a significant benefit in OS and DFS for p16-positive OPSCC patients. Our most interesting result is the significant benefit in OS and DFS for p16-positive patients when treated with RT+EGFR inhibitors compared with conventional RT+CT (p = 0.01), whereas this benefit was not observed in p16-negative patients (p = 0.9). From 2000 to 2011, more than 304 patients were treated in our center with RT plus platinum-based CT. To ensure this study provided results from a representative sample of our patients, we assessed the OS rates of the entire group. The median OS of these 304 patients was 37 months (range of 21–53 months), with 2- and 5-year OS rates of 56% and 37%, respectively. These results are similar to those observed for the group of 108 patients analyzed in this study, who had a median OS of 32 months (range of 18–46 months) and 2- and 5-year OS rates of 58% and 35%, respectively.

We found HPV16 DNA in 11% of the patients and in 15% of the OPSCCs. Both of these prevalences are lower compared with data from a recent meta-analysis (22-34% in HNSCC and 30-41% in OPSCC) [8,10,25], possibly due to the epidemiologic profile of our population, which had a high proportion of former/current and heavy tobacco users.

Several studies have confirmed a better outcome for patients with HPV-positive HNSCC compared with HPV-negative patients. The majority of these studies have been performed in OPSCC, so this benefit is well established in this subset of tumors [13,14,17-21]. A recent meta-analysis of 34 studies published by Dayyani et al. has shown a benefit in OS for HPV-positive patients compared to HPV-negative patients (HR = 0.4; 95% CI 0.2 to 0.6; p = 0.0001), including all locations of HNSCC [25]. According with these data, several studies have shown that among patients with HNSCC and OPSCC managed with different treatment modalities, those with p16-positive tumors have a better prognosis in terms of response, recurrence and survival than patients with p16-negative tumors [14,22-24]. Recently, the results of a phase III clinical trial (TROG 02.02) with concurrent RT+CT with or without tirapazamine has shown promising results in the subgroup of patients with OPSCC using p16 immunohistochemistry as a prognostic factor. Patients with p16-positive tumors had better 2-year OS and failure-free survival (FFS) rates compared with patients with p16-negative tumors (OS 91% vs. 74%; p = 0.04; FFS 87% vs. 72%; p = 0.003). Cox regression analysis of OS, including the prognostic factors of hemoglobin, T category, N category, and ECOG performance status, demonstrated that p16 status was the only significant factor in multivariable analysis (HR = 0.4; 95% CI 0.2 to 0.9; p = 0.03) [24].

The HPV-related beneficial outcome in this neoplasm indicates that these tumors respond well to conventional treatment approaches. The biologic basis for this observation is under investigation, but it could be due to some extent to an increased sensitivity to RT+CT of functional, non-mutated p53 [26] and the absence of field cancerization related to tobacco/alcohol exposure. Moreover, an increased sensitivity to apoptosis has been observed in E6/E7-positive human keratinocytes when exposed to cisplatin-based CT [4,27]. To the best of our knowledge, only one study has reported differences in survival in patients treated with EGFR inhibitors according to p16 or HPV16 status [28] That phase II trial, which applied induction CT based on paclitaxel, carboplatin and cetuximab followed by either RT, concomitant RT+CT, or surgery, for locally advanced (LA)-HNSCC, shows a robust benefit in survival for HPV-positive patients over HPV-negative patients, with improved progression-free survival (p = 0.012) and OS (p = 0.046). The current study supports this better outcome for HPV-positive patients regardless the treatment received.

Our study found a better outcome in terms of DFS and OS in p16-positive patients treated with RT+EGFR inhibitors compared with those treated with conventional RT+CT. The reason for these findings remains unclear. An inverse relationship between HPV status and EGFR expression has been demonstrated in several recent head and neck cancer studies [15,29-31]. Most of them suggest that a combination of HPV and EGFR may more accurately predict the outcome of patients than either alone. One recent study examined the prognostic significance of EGFR in relation to HPV in a large cohort of patients with OPSCC. That study reported that HPV was a predictor of loco-regional recurrence, event-free survival, and OS after adjustment for clinicopathological variables, including EGFR. This study showed that HPV-negative/EGFR-positive patients had an adjusted 13-fold increased risk of having a loco-regional failure and more than a 4-fold increased risk of dying compared with HPV-positive/EGFR-negative patients [30]. Although several preclinical studies have shown that HPV16 E6 and E7 expression sensitizes human keratinocytes to apoptosis caused by cisplatin, etoposide and mitomycin C by mechanisms that are not fully understood [27], little information is available regarding EGFR inhibitors and HPV-related cell tumors.

The five-year survival data of the study of Bonner et al. on RT plus cetuximab for locoregionally advanced head and neck cancer show some interesting associations [32]. Cetuximab seemed to provide the most benefit for patients with oropharyngeal tumors, T1–3 tumors, advanced nodal stage, and high Karnofsky performance status, characteristics associated with HPV-related tumors. These subgroups represent small numbers of patients and might represent spurious findings, so further work should be performed to test the consistency of these results.

Our study has some limitations, such as the retrospective analysis, the small sample size, the limited number of p16-positive patients (18) and the fact that p16 expression was not an independent prognostic factor in the multivariable analysis. The retrospective setting and the possibility of selection bias may be considered pitfalls, and although patient characteristics between treatment groups were not completely well balanced (more women and less alcohol consumption in the group treated with RT+EFGR inhibitors), we should point that the most important clinicopathological variables (tumor size, N category and ECOG performance status) were well distributed between both arms of treatment. The issue of sample size is another limitation because the number of p16-positive patients in each arm of treatment was small (8 RT+EGFR and 10 RT+CT), but despite the limited sample size, the survival benefit in p16-positive patients treated with RT+EGFR inhibitors compared with RT+CT reached statistical significance.

## Conclusions

In conclusion, this study provides the first evidence to support a better outcome for p16-positive HNSCC patients when treated with RT combined with EGFR inhibitors vs. RT combined with traditional cisplatin-based CT. It is necessary to conduct specific clinical trials for p16-positive patients to determine if RT combined with EGFR inhibitors is the optimal approach in this growing population. Several studies are ongoing.

## Abbreviations

CR: Complete response; DFS: Disease-free survival; EGFR: Epidermal growth factor receptor; FFPE: Formalin-fixed, paraffin-embedded; FFS: Failure-free survival; HNSCC: Head and neck squamous cell carcinoma; HPV: Human papillomavirus; IHC: Immunohistochemistry; LA-HNSCC: Locally advanced head and neck squamous cell carcinoma; OPSCC: Oropharyngeal squamous cell carcinoma; OS: Overall survival; p16INK4A: p16; PCR: Polymerase chain reaction; RT: Radiotherapy; ST: Second tumors

Presented at American Society of Clinical Oncology meeting, June 2011. Poster discussion - abstract 80375.

## Competing interest

All authors have no conflicts of interest.

## Authors’ contributions

All authors read and approved the final manuscript.

## Grant support

We thank the Andalusian Cancer Society for its financial support.

## Pre-publication history

The pre-publication history for this paper can be accessed here:

http://www.biomedcentral.com/1471-2407/13/26/prepub
